# Size-fractionated microbiome observed during an eight-month long sampling in Jiaozhou Bay and the Yellow Sea

**DOI:** 10.1038/s41597-022-01734-3

**Published:** 2022-10-07

**Authors:** Jianchang Tao, Wenxiu Wang, JL Weissman, Yongyu Zhang, Songze Chen, Yuanqing Zhu, Chuanlun Zhang, Shengwei Hou

**Affiliations:** 1 Shenzhen Key Laboratory of Marine Archaea Geo-Omics, Department of Ocean Science and Engineering, Southern University of Science and Technology, Shenzhen, 518000 China; 2 Southern Marine Science and Engineering Guangdong Laboratory (Guangzhou), Guangzhou, 511458 China; 3 Shanghai Sheshan National Geophysical Observatory, Shanghai Earthquake Agency, Shanghai, 200062 China; 4 Department of Biological Sciences-Marine and Environmental Biology, University of Southern California, Los Angeles, CA 90089 USA; 5 Research Center for Marine Biology and Carbon Sequestration, Shandong Provincial Key Laboratory of Energy Genetics, Qingdao Institute of Bioenergy and Bioprocess Technology, Chinese Academy of Sciences, Qingdao, 266000 China; 6 State Key Laboratory for Marine Environmental Science, Institute of Marine Microbes and Ecospheres, Xiamen University, Xiamen, 361102 China

**Keywords:** Microbial ecology, Marine biology

## Abstract

Jiaozhou Bay is a typical semi-enclosed bay with a temperate climate imposed by strong anthropogenic influence. To investigate microbial biodiversity and ecosystem services in this highly dynamic coastal environment, we conducted a monthly microbial survey spanning eight months at two stations in the bay and the open Yellow Sea starting in April 2015. This report provides a comprehensive inventory of amplicon sequences and environmental microbial genomes from this survey. In total, 2,543 amplicon sequence variants were obtained with monthly relative abundance profiles in three size fractions (>2.7 μm, 2.7–0.7 μm, and 0.7–0.22 μm). Shotgun metagenomes yielded 915 high-quality metagenome-assembled genomes with ≥50% completeness and ≤5% contamination. These environmental genomes comprise 27 bacterial and 5 archaeal phyla. We expect this comprehensive dataset will facilitate a better understanding of coastal microbial ecology.

## Abstract (summary)

**Table:** 

Measurement(s)	temperature of seawater • salinity of seawater • chlorophyll a • particulate organic carbon (POC) of seawater • dissolved organic carbon (DOC) of seawater • total nitrogen (TN) of seawater • total nitrate of seawater • nitrite of seawater • Phosphate of seawater • Silicate of seawater • Ammonium of seawater
Technology Type(s)	CTD sampler (Sea-Bird) • Shimadzu TOC-VCPH analyzer • Shimadzu TOC-L Analyzer • SEAL AutoAnalyzer 3 automatic continuous flow analyzer
Factor Type(s)	geographic location • temporal instant
Sample Characteristic - Organism	marine metagenome
Sample Characteristic - Environment	marine seawater
Sample Characteristic - Location	Jiaozhou Bay

## Background & Summary

The global importance of microorganisms in biogeochemical cycling and climate change has been widely recognized^1^. Microbes play a central role in the marine food web by mediating carbon flow to upper trophic levels^2^, and are proposed to be responsible for the massive accumulation of recalcitrant dissolved organic carbon (rDOC) in the global ocean^3,4^. Seawater contains a contiguous body of particles, which have a predominant influence on microbial community assemblages. For instance, microbial community composition on particles was found to be consistently similar throughout the water column on a global scale^5^, and the size of particles may also play an important role in shaping microbial assemblage and community functioning^6^.

Particle niche partitioning suggests different trophic strategies. Free-living microbial communities are repeatedly observed to be distinct from particle-associated assemblages in both epipelagic^7–9^ and bathypelagic oceans^10,11^. Microbes colonizing particles were found to be phylogenetically conserved^11^ and metabolically more active than free-living ones^12,13^. Although there are plenty of studies showing that the rapid community response to particulate organic matters or nutrients is frequently associated with an altered microbial life strategy^14–17^, it is also evident that ecological interactions can complicate the interpretation^18^, particularly at finer phylogenetic resolutions^19^. Thus, it would be desirable to gain a better understanding of microbial particle association in both evolutionary and ecological aspects.

**Fig. 1:**
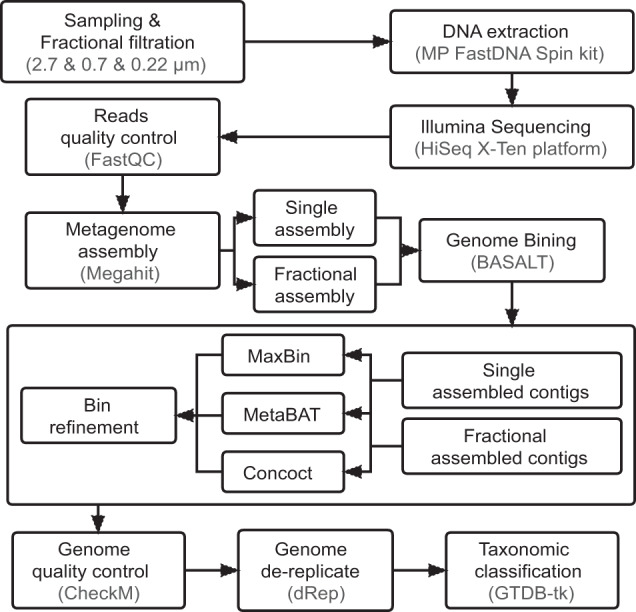
Bioinformatics workflow for amplicon and metagenomic data analysis. After sampling and filtration, microbial genomic DNA was extracted and libraries were prepared for amplicon and metagenomic sequencing. For amplicon data analysis, reads were quality controlled, denoised, and clustered using plugins of the QIIME2 suite. For metagenomic data analysis, preliminary MAGs were obtained after read quality control, metagenomic assembly and binning, then subjected to genome refinement and dereplication. The final MAGs were taxonomically classified and were used for further analysis. Detailed data processing steps, software, and parameters can be found in the Methods section.

**Fig. 2:**
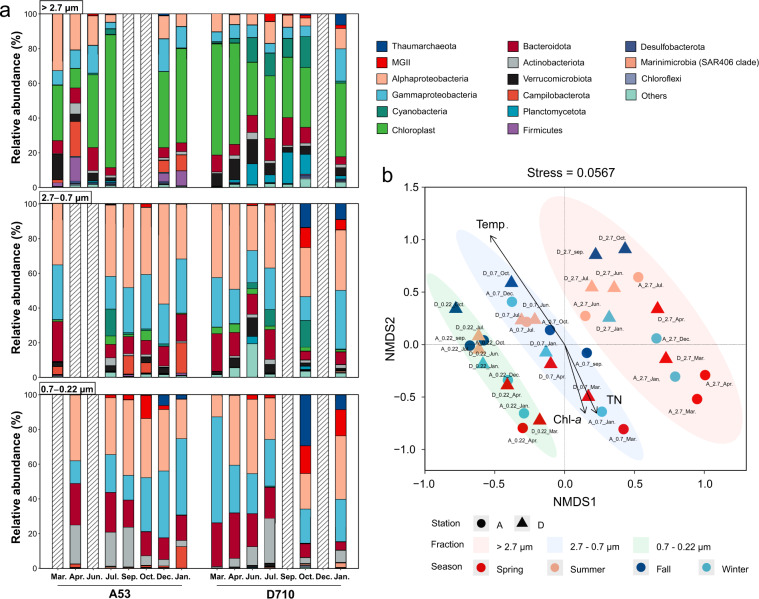
Relative abundance of ASVs in three size fractions (**a**) and NMDS ordination of samples based on Bray-Curtis dissimilarity matrix (**b**). ASVs were color-coded according to their phyla, and those with total relative abundances of <5% in all 37 samples were grouped into Others. Hatched bars represent missing samples. Environmental factors were fit to the ordination using the envfit function in the vegan R package. Only factors with a significance level of <0.05 were shown. Detailed abundance data can be found in Supplementary Table 2, and associated environmental factors can be found in Supplementary Table 1.

Coastal and estuarine environments are dynamic systems suffering from multiple anthropogenic stresses. Jiaozhou Bay (JZB) is such a typical semi-enclosed inlet of the Yellow Sea under strong and long-lasting human impact. Substantial allochthonous organic matter is transported into the bay via several adjoining rivers, in addition to particles and pollutants released by a broad range of marine aquaculture farms^20,21^, causing eutrophication and frequent seasonal algal blooms in the past decades^22^. A systematic survey of microbial communities associated with different sizes of particles is required to better understand microbial driven biogeochemical cycles in this specific system. Here we carried out a monthly microbial sampling starting from April 2015 for eight months at two stations, in the bay (station A53) and the connected open Yellow Sea (station D710) (Fig. S1). Detailed sample metadata including environmental factors can be found in the supplementary Table 1. Seawater samples were consecutively filtered through three pore-sized filters to collect microorganisms colonizing in the corresponding fractions (Fig. 1). After DNA extraction, samples collected in September 2015 and March 2016 were subjected to metagenomic sequencing, and samples collected in the first 8 months were subjected to amplicon sequencing (Fig. 1). Eukaryotic phytoplankton as approximated by chloroplast 16S rDNA sequences dominated the >2.7 μm size fraction (11%–76%), though cyanobacteria (mainly *Synechococcus*) could occasionally accounted for :17% of all 16S rDNA reads in late summer (Fig. 2a). Alphaproteobacteria and Gammaproteobacteria were two most abundant phyla in the <2.7 μm size fractions (Fig. 2a). Bacteroidota and Actinobacteriota dominated the 0.7–0.22 μm fraction, each accounting for 4.7%–26% and 3.5%–23% of total amplicon reads on average, respectively (Fig. 2a). Although one should realize that the filtration cutoff does not provide an exact size exclusion since smaller microbes could be clogged in filters of the larger fraction, here microbial communities could be mainly partitioned into three clusters corresponding to the size fraction they primarily occupied as shown in the NMDS analysis (Fig. 2b). And all the three clusters were significantly influenced by temperature, Chl-*a*, and TN (total nitrogen) (*p* < 0.05, Fig. 2b).

**Fig. 3:**
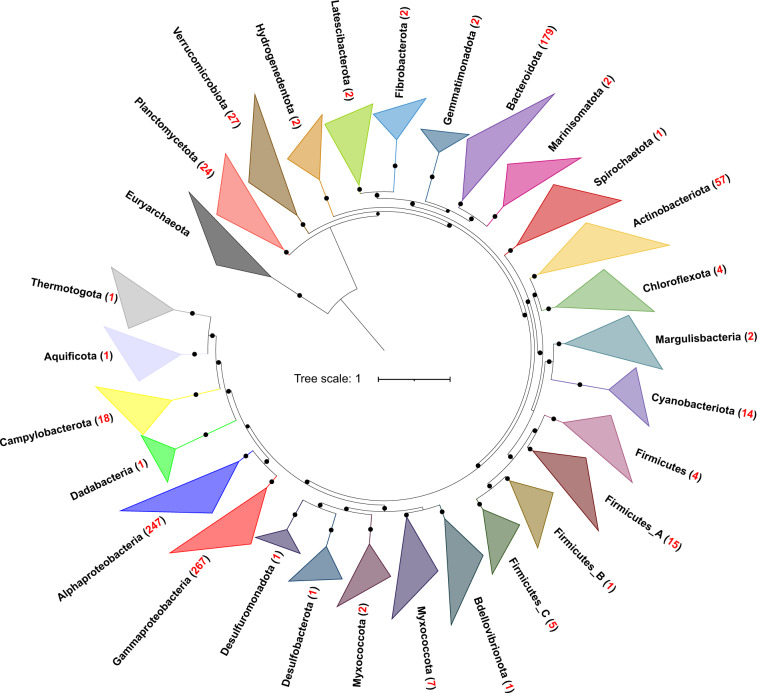
Phylogenomic placement of JZB bacterial MAGs. The maximum likelihood tree was reconstructed based on the concatenated alignments of 119 single-copy marker genes extracted from 890 JZB bacterial MAGs and 2656 reference genomes. The total number of MAGs recovered for each phylum was given in the parenthesis after the phylum name. Nodes with bootstrap values >0.5 were labeled in the dendrogram using filled black circles with sizes proportional to the validity from 0.5 to 1. Five archaeal genomes in the Euryarchaeota phylum were used as the outgroup to root the tree. Detailed MAG taxonomy assignment, associated with completeness and contamination information can be found in Supplementary Table 3.

**Fig. 4:**
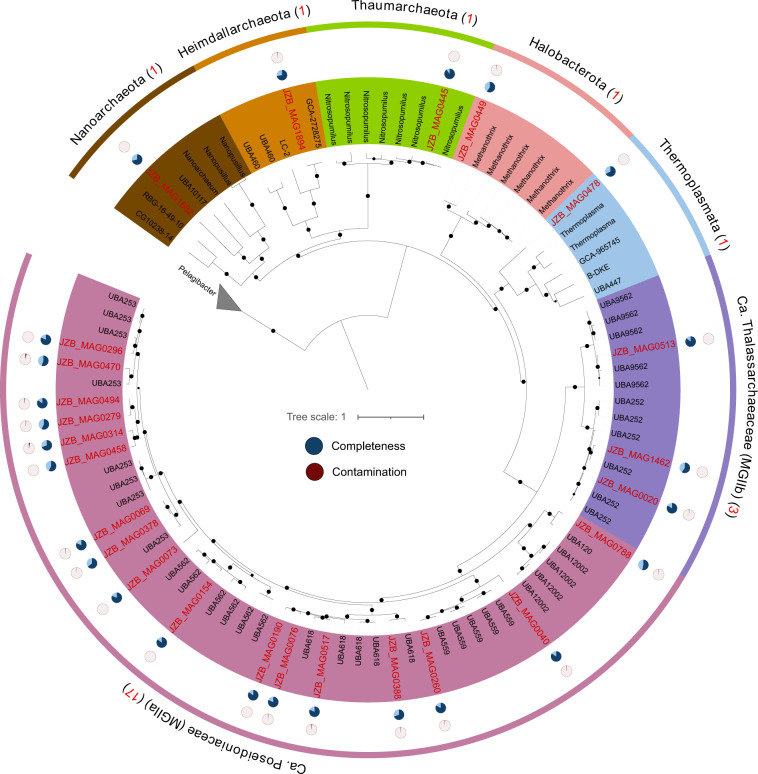
Phylogenomic placement of JZB archaeal MAGs with completeness and contamination information. The maximum likelihood tree was reconstructed based on the concatenated alignments of 117 single-copy marker genes extracted from 25 JZB archaeal MAGs (in red) and 68 reference genomes (in black). The total number of MAGs recovered for each phylum was given in the parenthesis after the phylum name. Nodes with bootstrap values >0.5 were labeled in the dendrogram using filled black circles with sizes proportional to the validity from 0.5 to 1. Five *Pelagibacter* genomes were used as the outgroup to root the tree. Detailed MAG taxonomy assignment, associated with completeness and contamination information can be found in Supplementary Table 3.

Metagenomes were both assembled individually or co-assembled for each size fraction, and 915 non-redundant and purified environmental metagenomic-assembled genomes (MAGs) with ≥50% completeness and ≤5% contamination were obtained (Supplementary Table 3: MAGs info.), comprising 27 bacterial and 5 archaeal phyla (Figs. 3, 4). Among these MAGs, 469 have a completeness score of ≥75%, and 183 are near complete (≥90%). Bacterial MAGs were mainly from Proteobacteria (247 MAGs belonging to 20 orders of Alphaproteobacteria and 267 MAGs belonging to 21 orders of Gammaproteobacteria), Bacteroidota (179 MAGs out of 8 orders), and Actinobacteriota (57 MAGs out of 8 orders) (Fig. 3). Archaeal MAGs were mainly Marine Group II archaea (MGII), include 17 MGIIa MAGs and 3 MGIIb MAGs (Fig. 4). All the 14 MAGs of Cyanobacteriota are in the order of Synechococcales A, 11 of them are *Synechococcus*, and 3 are *Cyanobium*.

This monthly microbial survey at two contrasting stations provides a comprehensive and valuable database for studying microbial community succession and nutrient cycling of coastal marine environments.

## Methods

### Sampling and physicochemical analyses

Samples were collected at two stations (Fig. S1) in 2015 (April, June, July, September, and December) and 2016 (January, March, and October) in JZB and the Yellow Sea on the “Innovation” research vessel operated by the Institute of Oceanography, Chinese Academy of Sciences. Microbial cells were collected onto three different pore-sized filters, e.g., 2.7 μm (Whatman GF/D 1823-047), 0.7 μm (Whatman GF/F 1825-047) and 0.22 μm (Millipore GTTP04700), by sequentially filtering 4-L seawater collected at 0.5 m depth using a CTD sampler (Sea-Bird). Additionally, 1-L seawater was directly filtered through a 0.7 μm pore-sized filter for particulate organic carbon (POC) measurement using a Shimadzu TOC-VCPH analyzer, and 50-ml seawater was sampled to measure the concentrations of dissolved organic carbon (DOC) and total nitrogen (TN) using a Shimadzu TOC-L Analyzer^23^. All filters and water samples were stored at −20 °C. Environmental parameters, including water temperature, salinity, and Chl-*a* concentration, were measured by the conductivity-temperature-depth (CTD) probe on board. Concentrations of specific nutrients, including the total nitrate, $N{O}_{2}^{-},P{O}_{4}^{3-},Si{O}_{3}^{2-}$, and $N{H}_{4}^{+}$, were measured using a SEAL AutoAnalyzer 3 automatic continuous flow analyzer in the lab.

### DNA extraction and metagenomic sequencing

Samples taken in September 2015 and March 2016 were also selected for metagenomic sequencing to investigate microbial community disturbances during marine aquaculture farming and spring algal blooms. DNA was extracted for filter samples taken from the two months using FastDNA SPIN for soil kit (MP Biomedicals, LLC) according to the user manual. DNA materials were then sheared by a Covaris M220 Focused-ultrasonicator (Covaris, Woburn, MA, United States), and the resulting ~350 bp long fragments were further purified using MinkaGene Gel Extraction Kit (mCHIP, Guangzhou, China). Illumina libraries were constructed from about 100 ng DNA using NEB Next UltraTM DNA Library Prep Kit for Illumina (New England Biolabs, United States) according to the manufacturer’s instructions. Sequencing was performed on an Illumina Hiseq X-Ten platform using the 2 × 150 bp paired-end chemistry at Magigene Biotechnology (Guangzhou, China).

### 16S rDNA sequencing

The V4 variable regions of 16S rDNA were amplified for 37 samples using the pair of universal prokaryotic primers 515FB (GTGYCAGCMGCCGCGGTAA) and 806RB (GGACTACNVGGGTWTCTAAT)^24^. The PCR mixture contained 25 μl of 2× Premix Taq DNA polymerase (TaKaRa), 0.2 mM of each primer, 20 μl of ddH2O, and 3 μl of template DNA in a total volume of 50 μl. Thermocycling steps were as follows: an initial denaturation step for 30 s at 94 °C, followed by 30 amplification cycles of 94 °C for 30 s, 58 °C for 30 s and 72 °C for 30 s, and a final elongation step at 72 °C for 30 s. Indexed PCR products were pooled and purified using the EZNA Gel Extraction Kit (Omega, USA) to remove primer dimers, and sequenced on the MiSeq platform (2 × 300 PE, Illumina) at MajorBio Biotechnology (Shanghai, China). Raw reads were analyzed using the Quantitative Insights into Microbial Ecology (QIIME2, version 2020.8) software suite with the demux, DADA2 and feature-table plugins^25^. Features with a total abundance of less than 10 across all samples, or those only present in one sample were discarded. Since sequencing depth was sufficient (Fig. S2), we subsampled the sequencing depth to the minimum sequence number of all samples, and reported a final ASV (Amplicon Sequence Variant) abundance table with an even depth across samples (Supplementary Table 2). ASV taxonomy was assigned by the feature-classifier plugin using Naive Bayes against the SILVA v138 99% dereplicated reference database (https://www.arb-silva.de/ngs).

### Microbial community clustering analysis

Non-metric multidimensional scaling (NMDS) based on Bray-Curtis distance was used to compare the differences in microbial community composition across samples, and environmental factors were fitted to the NMDS axes using the envfit method with 999 Monte Carlo tests using the vegan R package. Only factors with a significance level of <0.05 were included in the NMDS figure (Fig. 2b).

### Sequence quality control and metagenomic assembly

HiSeq generated raw reads were first trimmed by Trim Galore v0.5 using default settings to remove adaptors and low quality (below Q20) regions, and the final read quality was assessed using FastQC v0.11.8. Trimmed reads longer than 20 bp were used as clean reads for downstream analyses. All 10 metagenomic samples collected in September 2015 and March 2016 were assembled individually using megahit v.1.1.3^26^, with the following parameters:–presets meta-sensitive and–min-count 1–k-list 25, 29, 39, 49, 59, 69, 79, 89, 99, 109, 119, 129, 141. In addition, a co-assembly step was done for samples from the same size fraction. Final assemblies were evaluated using Quast v.4.6.3^27^.

### MAG generation, refinement, purification, and taxonomy assignment

Individually assembled and co-assembled contigs longer than 1 kb were subjected to metagenomic binning using BASALT^28^, which employed MetaBAT v2.12.1^29^, Maxbin v2.2.4^30^ and CONCOCT v1.1.0^31^ to make original bins, then compared these raw bins across assemblies to obtain a set of refined non-redundant MAGs. In addition, these MAGs were further refined using MAGpurify v2.12^32^ to remove contaminations using the “phylo-markers”, “tetra-freq”, “gc-content”, “known-contam” and “clade-markers” modules. Genome quality was assessed using CheckM v1.0.11^33^, and MAGs with completeness higher than 50% and contamination lower than 5% were further dereplicated using dRep v2.6.2^34^ at 98% identity. The quality of MAGs was assessed by prok-quality^35^ following the MIMAG standards^36^. Taxonomies of dereplicated MAGs were assigned using GTDB-Tk v0.1.6 based on the GTDB v86 database^37,38^.

### Phylogenomic tree construction

Universal bacterial or archaeal single-copy marker genes used by the GTDBtk v1.7 were identified and extracted from both JZB MAGs and closest relative genomes in the GTDB v89 database. Only marker genes found in ≥30 genomes were selected to build the bacterial and archaeal phylogenomic trees. Protein sequences of selected marker genes were first aligned using MUSCLE (v3.8.31)^39^, and then trimmed using trimAl (v1.4)^40^ with the “-automated1” option. The concatenated alignment sequences were used as the input of FastTree v2.1.1^41^ to build phylogenomic trees with the “-gamma-lg” option. And the resulting trees were visualized using iTOL online server (https://itol.embl.de).

## Data Records

Raw reads of 16S rDNA and metagenome generated in this study have been deposited in the National Center for Biotechnology Information BioProject database with the project ID PRJNA823870^42^ and PRJNA823908^43^. Contigs, MAGs and supplementary files have been deposited at figshare^44^.

## Technical Validation

All raw data processing steps, software and parameters used in this study were described in the Methods section. The quality of the genomes was also assessed by CheckM, and genomic statistics can be found in the supplementary tables.

## Supplementary information

Supplementary Table 1

Supplementary Table 2

Supplementary Table 3

Figure S1

Figure S2

## Data Availability

All versions of third-party software and scripts used in this study are described and referenced accordingly in the Methods sections for ease of access and reproducibility.
