# Publisher Correction: Size-fractionated microbiome observed during an eight-month long sampling in Jiaozhou Bay and the Yellow Sea

**DOI:** 10.1038/s41597-022-01750-3

**Published:** 2022-10-13

**Authors:** Jianchang Tao, Wenxiu Wang, JL Weissman, Yongyu Zhang, Songze Chen, Yuanqing Zhu, Chuanlun Zhang, Shengwei Hou

**Affiliations:** 1 Shenzhen Key Laboratory of Marine Archaea Geo-Omics, Department of Ocean Science and Engineering, Southern University of Science and Technology, Shenzhen, 518000 China; 2 Southern Marine Science and Engineering Guangdong Laboratory (Guangzhou), Guangzhou, 511458 China; 3 Shanghai Sheshan National Geophysical Observatory, Shanghai Earthquake Agency, Shanghai, 200062 China; 4 Department of Biological Sciences-Marine and Environmental Biology, University of Southern California, Los Angeles, CA 90089 USA; 5 Research Center for Marine Biology and Carbon Sequestration, Shandong Provincial Key Laboratory of Energy Genetics, Qingdao Institute of Bioenergy and Bioprocess Technology, Chinese Academy of Sciences, Qingdao, 266000 China; 6 State Key Laboratory for Marine Environmental Science, Institute of Marine Microbes and Ecospheres, Xiamen University, Xiamen, 361102 China

**Keywords:** Microbial ecology, Marine biology, Microbial ecology, Marine biology

Correction to: *Scientific Data* 10.1038/s41597-022-01734-3, published online 07 October 2022

The original version of this Article contained an error in the spelling of the author JL Weissman, which was incorrectly given as J L Weissman. This has now been corrected in both the PDF and HTML versions of the Article.

